# Impact of timing of continuous intravenous anesthetic drug treatment on outcome in refractory status epilepticus

**DOI:** 10.1186/s13054-018-2235-2

**Published:** 2018-11-21

**Authors:** Dominik Madžar, Caroline Reindl, Antje Giede-Jeppe, Tobias Bobinger, Maximilian I. Sprügel, Ruben U. Knappe, Hajo M. Hamer, Hagen B. Huttner

**Affiliations:** Friedrich-Alexander University Erlangen-Nürnberg (FAU), Department of Neurology, Schwabachanlage 6, 91054 Erlangen, Germany

**Keywords:** Refractory status epilepticus, Status Epilepticus Severity Score, Continuous intravenous anesthetic drugs, Anesthetics, Outcome

## Abstract

**Background:**

Patients in refractory status epilepticus (RSE) may require treatment with continuous intravenous anesthetic drugs (cIVADs) for seizure control. The use of cIVADs, however, was recently associated with poor outcome in status epilepticus (SE), raising the question of whether cIVAD therapy should be delayed for attempts to halt seizures with repeated non-anesthetic antiepileptic drugs. In this study, we aimed to determine the impact of differences in therapeutic approaches on RSE outcome using timing of cIVAD therapy as a surrogate for treatment aggressiveness.

**Methods:**

This was a retrospective cohort study over 14 years (*n* = 77) comparing patients with RSE treated with cIVADs within and after 48 h after RSE onset, and functional status at last follow-up was the primary outcome (good = return to premorbid baseline or modified Rankin Scale score of less than 3). Secondary outcomes included discharge functional status, in-hospital mortality, RSE termination, induction of burst suppression, use of thiopental, duration of RSE after initiation of cIVADs, duration of mechanical ventilation, and occurrence of super-refractory SE. Analysis was performed on the total cohort and on subgroups defined by RSE severity according to the Status Epilepticus Severity Score (STESS) and by the variables contained therein.

**Results:**

Fifty-three (68.8%) patients received cIVADs within the first 48 h. Early cIVAD treatment was independently associated with good outcome (adjusted risk ratio [aRR] 3.175, 95% confidence interval [CI] 1.273–7.918; *P* = 0.013) as well as lower chance of both induction of burst suppression (aRR 0.661, 95% CI 0.507–0.861; *P* = 0.002) and use of thiopental (aRR 0.446, 95% CI 0.205–0.874; *P* = 0.043). RSE duration after cIVAD initiation was shorter in the early cIVAD cohort (hazard ratio 1.796, 95% CI 1.047–3.081; *P* = 0.033). Timing of cIVAD use did not impact the remaining secondary outcomes. Subgroup analysis revealed early cIVAD impact on the primary outcome to be driven by patients with STESS of less than 3.

**Conclusions:**

Patients with RSE treated with cIVADs may benefit from early initiation of such therapy.

**Electronic supplementary material:**

The online version of this article (10.1186/s13054-018-2235-2) contains supplementary material, which is available to authorized users.

## Introduction

There is an ongoing debate on the risks and benefits of the use of continuous intravenous anesthetic drugs (cIVADs) for treatment of refractory status epilepticus (RSE) [1]. As prolonged seizures have been linked to neuronal damage in animal models [2, 3] and to poor functional outcome in humans [4, 5], guidelines advocate cIVADs as third-line therapy for rapid termination of seizures [6]. Both the Neurocritical Care Society and the European Federation of Neurological Societies, however, acknowledge a shortage of data supporting this therapeutic approach [7, 8]. Recent studies reported a negative impact of cIVAD therapy on outcome in RSE [9–11]. These studies compared status epilepticus (SE) cohorts treated with and without cIVADs, whereas the factors associated with negative outcome specifically among patients with RSE treated with cIVADs have gained little attention by now. In the present study, we sought to examine the effects of timing of initiation of cIVADs on outcome in RSE by using a 48-h cutoff after RSE onset for definition of early and late use of cIVADs. As cIVADs were reported to be particularly hazardous when used for treatment of milder forms of SE [9], we also analyzed subgroups defined by RSE severity graded by the Status Epilepticus Severity Score (STESS) and the variables included in the STESS [12].

## Methods

### Patients and data collection

Patients were eligible for inclusion into the study if they received treatment for RSE on the neurological intensive care unit of our institution between January 2001 and January 2015 with continuous intravenous infusion of at least one of the following drugs (cIVADs): midazolam, propofol, thiopental, and ketamine. We used our electronic medical records and electroencephalography databases to gather information on demographics, RSE etiology, severity and duration, treatment, complications, and outcome. Demographics included gender, age at admission, and premorbid functional status. Data were assessed by two independent reviewers using a standardized data extraction form (DM and RUK). In case of disagreement, data were analyzed by a third reviewer (HBH) and consensus was found through discussion. For reasons of comparability with previous research, RSE caused by hypoxic encephalopathy was excluded. Only incidence episodes were considered; that is, in case of recurrent treatment with anesthetics for RSE in our institution during the study period, only the first episode was entered into the study [9].

### Refractory status epilepticus: definition, duration, and severity

In accordance with previous studies, SE was defined as clinically or electroencephalographically persisting seizure with duration of at least 5 min or as a series of seizures without interictal recovery [8]. The worst seizure semiology prior to initiation of antiepileptic therapy was used to categorize episodes as simple partial SE (SPSE), complex partial SE (CPSE), generalized convulsive SE (GCSE), or non-convulsive SE (NCSE) in coma [10]. RSE was defined as SE with ongoing seizure activity despite application of two adequately dosed antiepileptic drugs (AEDs) [13]. RSE duration after initiation of cIVAD therapy was defined as time between the beginning of cIVAD treatment and clear and enduring electroencephalographic or clinical seizure cessation or both. Durations of RSE after initiation of cIVADs as well as times on mechanical ventilation were estimated in days. Any portion of one day in RSE or on the respirator, respectively, was counted as one full day. RSE severity was graded with the STESS and was dichotomized into STESS of less than 3 (mild) and STESS of at least 3 (severe) as previously proposed [12]. RSE etiology was categorized in accordance with the guidelines of the International League Against Epilepsy into acute symptomatic, remote symptomatic, progressive symptomatic, and unknown etiology [14]. A potentially fatal etiology was defined when meeting the criteria introduced by Rossetti et al. [15].

### Definition of early and late cIVAD therapy

Treatment with cIVADs was defined as *early* when started within 48 h after RSE onset; otherwise, it was defined as *late*.

### Outcome measures and outcome definitions

The primary outcome of this study was functional status at last available follow-up graded by the modified Rankin Scale (mRS) [16]. Information on outcome was extracted from discharge summaries of rehabilitation facilities or own records if patients had represented to our hospital. Outcome was defined as *good* in case of complete recovery after RSE (that is, when the mRS at last available follow-up equaled the premorbid mRS) or, in case of new disability, when the mRS at last available follow-up was less than 3. Otherwise, outcome was defined as *poor.* Secondary outcomes were functional outcome at discharge (with *good* and *poor* defined identically as for the primary outcome), in-hospital mortality, RSE termination, induction of burst suppression, use of thiopental, duration of RSE after initiation of cIVADs, duration of mechanical ventilation, and occurrence of super-refractory SE (SRSE).

### Statistical analysis

Statistical analyses were performed by using SPSS Statistics 21.0 (http://www.spss.com). *P* values of less than 0.05 were considered statistically significant, and all tests used were two-sided. Baseline clinical data and RSE characteristics were compared by using Pearson chi-squared test or, where appropriate, Fisher’s exact tests for categorical data and Mann–Whitney *U* test for continuous variables. Crude and adjusted risk ratios for primary and secondary outcomes were estimated by Poisson regression with robust error variance. Multivariable analysis adjusted for STESS (as continuous variable) and a potentially fatal etiology. These calculations were performed on the overall cohort as well as subgroups defined by the STESS (cutoff 3 points) and the variables included in it (that is, age, a history of seizures, level of consciousness, and worst seizure type). Time-to-event outcomes were compared with the Kaplan–Meier method with hazard ratios estimated by using Cox proportional hazard analysis. Patients in whom RSE could not be terminated prior to discharge or death were censored from this analysis.

## Results

### Study population

**Fig. 1:**
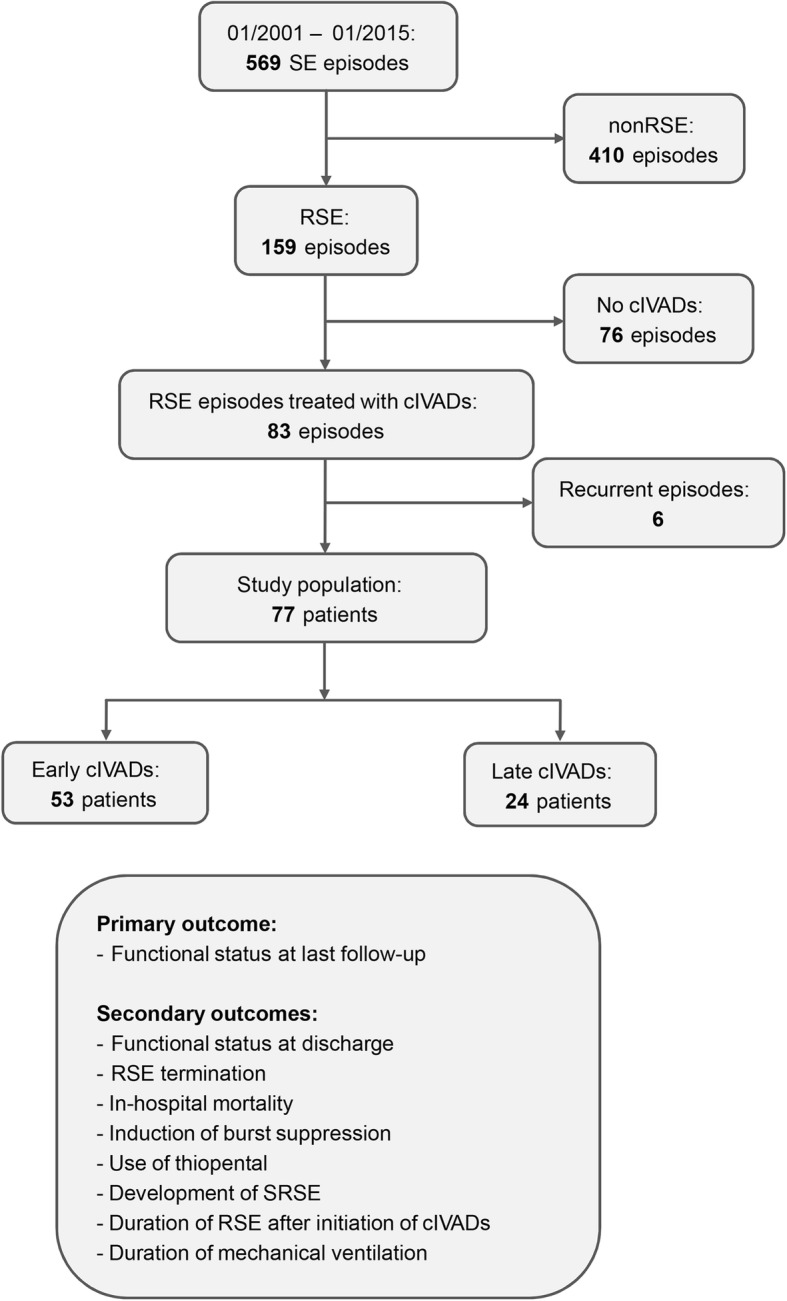
Flowchart of study cohort. In total, 569 episodes were treated for status epilepticus (SE) in our institution between January 2001 and January 2015. Among those, 159 satisfied the criteria of refractory SE. In 83 episodes, patients received treatment with continuous intravenous anesthetic drugs (cIVADs). Seven recurrent episodes were excluded, leaving 77 patients for final analysis, of whom 53 received early cIVADs and 24 received late cIVADs. Primary and secondary study outcomes are depicted. Abbreviations: *cIVAD* continuous intravenous anesthetic drug, *RSE* refractory status epilepticus, *SE* status epilepticus, *SRSE* super-refractory status epilepticus

We identified 159 RSE episodes in 131 patients. In 83 (52.2%) episodes, patients received treatment with cIVADs. After exclusion of recurrent episodes (*n* = 6), 77 patients remained for final analysis. Of those, 53 (68.8%) received cIVAD therapy within the first 48 h of RSE (Fig. 1). A description of the study cohort is presented in Table 1. Patients were treated with up to four cIVADs, but the majority received a maximum of two cIVADs (*n* = 59, 76.6%). The cIVAD most frequently used first was propofol (*n* = 47, 61.0%; Additional file 1: Table S1). In-hospital mortality was 24.7%, and RSE termination rate was 85.7%. Follow-up data were available for all but two patients (97.4%); one patient was lost to follow-up in the early and one in the late cIVAD cohort. The median follow-up time was 11 weeks (interquartile range [IQR] 6–25).

**Table 1:** Baseline characteristics of the study cohort

	Total cohort (*n* = 77)	Early cIVADs (*n* = 53)	Late cIVADs (*n* = 24)	*P* value
Demographics				
Gender, female	47 (61.0)	29 (54.7)	18 (75.0)	0.091
Age on admission, years	70 (52–76)	70 (49–77)	70 (54–76)	0.830
Premorbid mRS	2 (0–3)	2 (0–4)	2 (1–4)	0.928
Median STESS and STESS components				
STESS	3 (2–4)	3 (2–5)	2 (1–4)	0.106
History of seizures	40 (51.9)	25 (47.2)	15 (62.5)	0.212
Stuporous or comatose on admission	52 (67.5)	41 (77.4)	11 (45.8)	0.006
Complex partial SE	28 (36.4)	14 (26.4)	14 (58.3)	0.007
Generalized convulsive SE	34 (44.2)	30 (56.6)	4 (16.7)	0.001
NCSE in coma	15 (19.5)	9 (17.0)	6 (25.0)	0.535
Etiology				
Acute symptomatic	38 (49.4)	28 (52.8)	10 (41.7)	0.365
Remote symptomatic	18 (23.4)	11 (20.8)	7 (29.2)	0.419
Progressive symptomatic	10 (13.0)	5 (9.4)	5 (20.8)	0.270
Unknown	11 (14.3)	9 (17.0)	2 (8.3)	0.486
Potentially fatal	35 (45.5)	24 (45.3)	11 (45.8)	0.962

Data are number (percentage) or median (interquartile range)

Abbreviations: *cIVAD* continuous intravenous anesthetic drug, *mRS* modified Rankin Scale, *NCSE* non-convulsive status epilepticus, *SE* status epilepticus, *STESS* Status Epilepticus Severity Score

### Factors associated with cIVAD timing

Age, SE severity, and SE etiology did not differ significantly between episodes treated with and without early cIVADs (Table 1). In the early cIVAD group, impaired consciousness on admission and GCSE were more frequent while CPSE was rarer. A comparable number of patients had NCSE in coma. No patient had SPSE.

### Primary and secondary outcomes

The results of the analysis are summarized in Table 2. After adjustment for confounders, early use of cIVADs was an independent predictor of good outcome at last follow-up. The follow-up time did not differ significantly between the early and late cIVAD cohort (12 [IQR 6–26] versus 9 [IQR 5–18] weeks; *P* = 0.483).

**Table 2:** Overview of primary and secondary outcomes

	Early cIVADs (*n* = 53)	Late cIVADs (*n* = 24)	Unadjusted RR (95% CI)	*P* value for unadjusted RR	Adjusted RR^b^ (95% CI)	*P* value for adjusted RR
Primary outcome						
Good outcome at last follow-up	23 (44.2)^a^	4 (17.4)^a^	2.543 (0.992–6.521)	0.052	3.175 (1.273–7.918)	0.013
Secondary outcomes						
Good outcome at discharge	13 (24.5)	2 (8.3)	2.943 (0.720–12.037)	0.133	3.985 (0.874–18.161)	0.074
In-hospital mortality	14 (26.4)	5 (20.8)	1.268 (0.515–3.119)	0.605	1.086 (0.446–2.645)	0.856
RSE termination	45 (84.9)	21 (87.5)	0.970 (0.803–1.172)	0.970	0.980 (0.803–1.197)	0.980
Induction of burst suppression	32 (60.4)	22 (91.7)	0.659 (0.513–0.845)	0.001	0.661 (0.507–0.861)	0.002
Use of thiopental	9 (17.0)	11 (45.8)	0.370 (0.177–0.774)	0.008	0.446 (0.205–0.974)	0.043
Development of SRSE	30 (56.6)	18 (75.0)	0.775 (0.543–1.050)	0.095	0.769 (0.567–1.093)	0.153

^a^One episode lost to follow-up

^b^Adjusted for Status Epilepticus Severity Score and a potentially fatal etiology

Data in columns 2 and 3 are number (percentage)

Abbreviations: *CI* confidence interval, *cIVAD* continuous intravenous anesthetic drug, *RR* risk ratio, *RSE* refractory status epilepticus, *SRSE* super-refractory status epilepticus

**Fig. 2:**
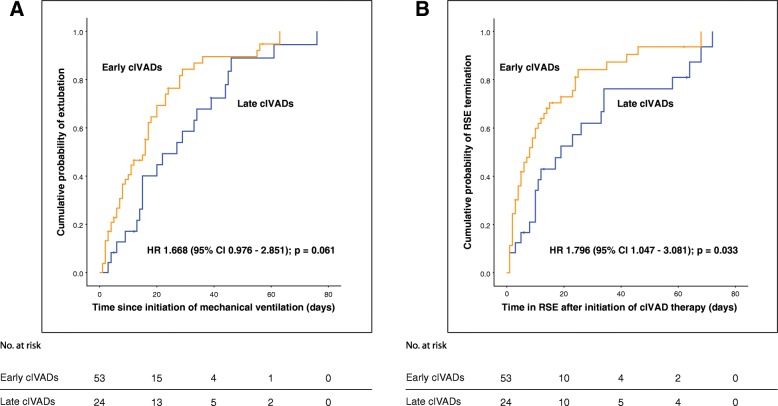
Kaplan–Meier curves for **a** duration of mechanical ventilation and **b** duration of status epilepticus after initiation of continuous intravenous anesthetic drugs. Censored cases are depicted by plus (+) marks along the curves. Abbreviations: *CI* confidence interval, *cIVAD* continuous intravenous anesthetic drug, *HR* hazard ratio, *RSE* refractory status epilepticus

In regard to secondary outcomes, early use of cIVADs was significantly associated with lower chance of both use of thiopental and induction of burst suppression but did not independently predict functional outcome at discharge, chance of RSE termination, risk of SRSE development, and in-hospital mortality. RSE duration after initiation of therapy was shorter in early cIVAD patients, whereas duration of mechanical ventilation did not differ significantly between cohorts (Fig. 2).

### Subgroup analysis for primary outcome

**Fig. 3:**
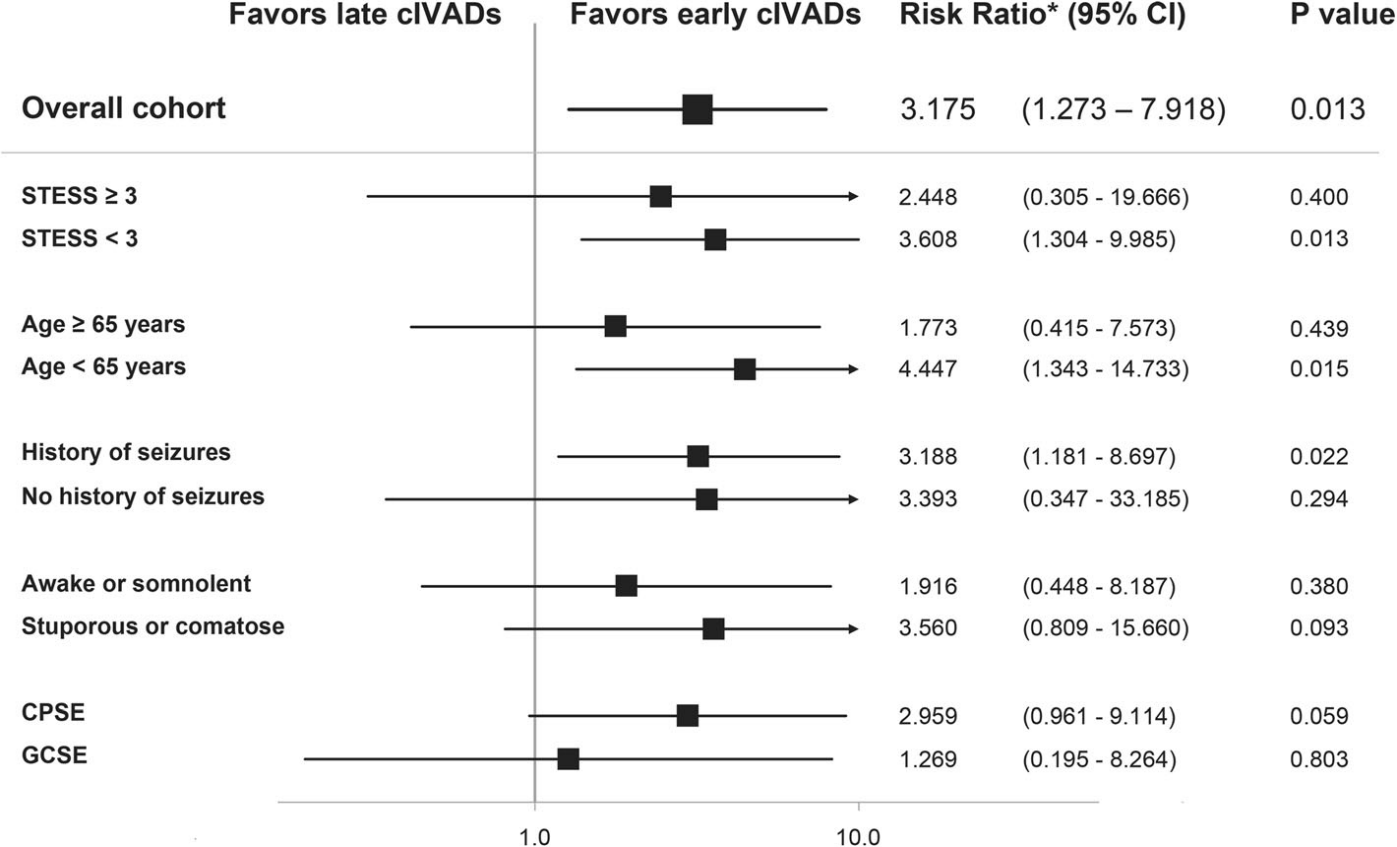
Poisson regression was used to compare the influence of early continuous intravenous anesthetic drug (cIVAD) therapy in subgroups defined by overall STESS as well as variables included in the STESS. *Risk ratios (RRs) were adjusted for STESS (as a continuous variable) and a potentially fatal etiology. In the subgroups of patients with non-convulsive status epilepticus (NCSE) in coma, one outcome had zero observations and therefore adjusted RRs could not be calculated. Abbreviations: *CI* confidence interval, *cIVAD* continuous intravenous anesthetic drug, *CPSE* complex partial status epilepticus, *GCSE* generalized convulsive status epilepticus, *STESS* Status Epilepticus Severity Score

The results of the subgroup analysis for the primary outcome are depicted in Fig. 3. Early use of cIVADs was independently associated with good outcome at last follow-up in patients with STESS less than 3, age less than 65 years, and a history of seizures but not in patients with STESS of at least 3, age of at least 65 years, and no previous seizures. Level of consciousness and worst seizure type did not influence impact of timing of cIVAD therapy on functional outcome. Because among patients with NCSE in coma one outcome had zero observations, calculation of adjusted risk ratios was not possible for this subgroup. The follow-up times did not differ significantly between patients who received cIVADs early compared with those who received treatment late in any of the subgroups (Additional file 2: Table S2).

## Discussion

In this study, we aimed to analyze the impact of timing of anesthetic therapy on the clinical course and the prognosis of RSE and found early initiation of cIVADs to be associated with higher chance of good outcome at last follow-up. This is one of only a few studies exclusively focusing on RSE episodes treated with continuous anesthetics, and several aspects of our results deserve attention.

First, we found reduced mental status and GCSE to be more frequent among patients who received treatment with cIVADs early. This probably reflects the fact that time of initiation of such therapy may be driven by clinical necessity rather than a specific treatment strategy in many cases, including those when patients have persisting convulsions after administration of two lines of non-anesthetic AEDs or require intubation for airway protection [17]. Both severely lowered levels of consciousness and generalized convulsive seizures are known predictors of negative outcome in SE [15, 18, 19]. Thus, although unfavorable prognosticators were more common among patients with early initiation of cIVADs, these individuals had better outcome. As there were no differences in baseline characteristics accounting for this fact, it appears plausible that this observation is related to the time of cIVAD initiation, especially because this factor was an independent predictor of outcome upon multivariable analysis.

Second, the choice of anesthetics administered requires discussion. Early cIVAD therapy was a negative predictor for the use of thiopental. Previous research found that, compared with other anesthetics, thiopental for RSE was associated with higher risk of complications, prolonged mechanical ventilation [20], duration of intensive care unit and overall hospital stay, and worse short- and long-term outcome [21]. Therefore, both clinical course and outcome could depend more on the choice of a specific anesthetic agent than on the time of initiation of cIVAD therapy. However, the decision to apply barbiturates may be influenced by a higher degree of seizure refractoriness suspected, especially as previous research found their use for RSE to be a surrogate for treatment aggressiveness [22]. Refractoriness to therapy in SE is known to increase with the duration of seizures [23] and therefore late use of anesthetics could lead to administration of a more hazardous agent, thus linking late use of cIVADs to poorer outcome.

Third, stratification of the study population according to the STESS and its components revealed that the positive effect of early cIVAD initiation in the overall cohort appeared driven primarily by the subgroups of individuals with STESS less than 3, age less than 65 years, and a history of seizures. A potential association between low RSE severity and good outcome following early cIVAD use deserves particular attention, given that previous reports advocated reserving aggressive therapy for patients scoring high on the STESS because of the risk-benefit ratio [12]. Our findings, however, support the opposite approach as rapid treatment escalation was related to positive outcome specifically among patients with RSE of low severity as indicated by a STESS less than 3. This finding is intriguing, but our results along with observations from previous studies offer a plausible explanation for it: (1) RSE duration was significantly shorter in the early cIVAD cohort in our study, (2) previous research found shorter RSE duration to be an independent predictor of positive outcome among RSE patients receiving cIVADs [24], and (3) low STESS frequently implies a more benign RSE etiology and therefore a larger impact of seizure duration on outcome [5].

To our knowledge, there are no studies that specifically aimed to examine the impact of cIVAD timing on outcome in RSE. In recent research on the effects of high versus low continuous intravenous midazolam (cIV-MDZ) infusion doses on RSE course and outcome, treatment was started significantly earlier in the high-dose cohort and these patients were less likely to die during hospital stay or to have withdrawal seizures; however, in a multivariable model, time of cIV-MDZ treatment was not an independent outcome predictor [25].

A secondary analysis of the Rapid Anticonvulsant Medication Prior to Arrival Trial (RAMPART) characterized the influence of early (defined as performed pre-hospital or within 30 min of emergency department arrival) versus late endotracheal intubation on outcome in SE and observed late intubation to be associated with higher mortality [26]. This finding points in the same direction as our observations, but, given major differences in inclusion criteria and in the definition of *early*, it is difficult to compare study results.

### Limitations

Our study has several clear limitations that need to be considered. The sample size is small, which is particularly problematic in a disorder as heterogenic as RSE. Data were collected retrospectively, and the medical records did not contain detailed enough information to determine the exact time of RSE cessation after initiation of cIVADs in all cases; therefore, we had to estimate RSE duration in days and not in hours. Furthermore, we could (with certainty) only tell *that* but not clearly assess the reasons *why* cIVADs were applied early or late. Some patients may have received cIVADs because they required intubation for airway protection or mechanical ventilation and not primarily for seizure control. Therefore, confounders not considered in our study may have substantially influenced therapeutic decisions and outcome, introducing the risk of bias into our results. Furthermore, we did not assess cIVAD dosing, and treatment of RSE with cIVADs did not follow a specific protocol in our institution during the early years of the study period. Setting the cutoff between early and late cIVAD treatment at 48 h after RSE onset represents an arbitrary definition which may appear inappropriate in light of guidelines advocating rapid treatment escalation. However, in our experience, a conservative approach in the first 48 h may represent a viable therapeutic option in non-convulsive RSE episodes, including those arising from GCSE either spontaneously or after initial treatment. The primary outcome measure of this study was not based on data collected at defined time points but relied on last available follow-up findings. Although follow-up durations were variable, they did not differ significantly between any of the cohorts compared. Furthermore, the validity of the primary outcome measure is supported by a trend toward higher chance of better outcome in the early cIVAD cohort already at time of discharge.

## Conclusions

Our findings indicate that when cIVADs are applied in RSE, prescribing them early may positively impact outcome, probably by shorter seizure duration and apparently mainly in those patients who do not have a severe RSE etiology dominating their prognosis. However, whether patients with RSE generally benefit from an aggressive or a conservative therapeutic approach cannot be answered by this study and this is because we exclusively focused on individuals treated with cIVADs.

## Additional files

Additional file 1: **Table S1.** Overview of continuous intravenous anesthetic drugs applied. (DOCX 14 kb)

Additional file 2: **Table S2.** Comparison of follow-up times between patients with early versus late continuous intravenous anesthetic drug (cIVAD) treatment in the subgroups defined by Status Epilepticus Severity Score (STESS) and its components. (DOCX 14 kb)

## Abbreviations

AED: Antiepileptic drug
CI: Confidence interval
cIVAD: Continuous intravenous anesthetic drug
cIV-MDZ: Continuous intravenous midazolam
CPSE: Complex partial status epilepticus
GCSE: Generalized convulsive status epilepticus
HR: Hazard ratio
IQR: Interquartile range
mRS: Modified Rankin Scale
NCSE: Non-convulsive status epilepticus
RR: Risk ratio
RSE: Refractory status epilepticus
SE: Status epilepticus
SPSE: Simple partial status epilepticus
SRSE: Super-refractory status epilepticus
STESS: Status Epilepticus Severity Score
