# Supplementary material for: Impact of timing of continuous intravenous anesthetic drug treatment on outcome in refractory status epilepticus
Source: Crit Care. 2018 Nov 21;22:317. doi: 10.1186/s13054-018-2235-2 (PMC6249897; doi:10.1186/s13054-018-2235-2)
Supplement: Supplementary file 1 — Table S1. Overview of continuous intravenous anesthetic drugs applied. (DOCX 14 kb) [file 13054_2018_2235_MOESM1_ESM.docx]

**Table S1:**

|  | **n (%)** |
| --- | --- |
| One cIVAD | 26 (33.8) |
| Two cIVADs | 33 (42.9) |
| Three cIVADs | 16 (20.8) |
| Four cIVADs | 2 (2.6) |
| Any propofol | 61 (79.2) |
| Any midazolam | 51 (66.2) |
| Any thiopental | 20 (26.0) |
| Any ketamine | 16 (20.8) |
| Only propofol | 17 (22.1) |
| Only midazolam | 9 (11.7) |
| Only thiopental | 0 |
| Only ketamine | 0 |
| Propofol first | 47 (61.0) |
| Midazolam first | 25 (32.5) |
| Thiopental first | 5 (6.4) |
| Ketamine first | 0 |

Abbreviations: cIVAD, continuous intravenous anesthetic drug
