# Supplementary material for: Impact of timing of continuous intravenous anesthetic drug treatment on outcome in refractory status epilepticus
Source: Crit Care. 2018 Nov 21;22:317. doi: 10.1186/s13054-018-2235-2 (PMC6249897; doi:10.1186/s13054-018-2235-2)
Supplement: Supplementary file 2 — Table S2. Comparison of follow-up times between patients with early versus late continuous intravenous anesthetic drug (cIVAD) treatment in the subgroups defined by Status Epilepticus Severity Score (STESS) and its components. (DOCX 14 kb) [file 13054_2018_2235_MOESM2_ESM.docx]

**Table S2:**

| **Subgroup** | **Early cIVADs** | **Late cIVADs** | **P value** |
| --- | --- | --- | --- |
| STESS ≥ 3 | 12 (7 – 17) | 9 (5 – 14) | 0.566 |
| STESS < 3 | 18 (3 – 38) | 10 (5 – 24) | 0.648 |
| Age ≥ 65 years | 12 (6 – 17) | 11 (6 – 24) | 0.875 |
| Age < 65 years | 11 (6 – 30) | 9 (5 – 19) | 0.527 |
| History of seizures | 14 (7 – 38) | 10 (5 – 24) | 0.614 |
| No history of seizures | 12 (5 – 17) | 9 (5 – 14) | 0.533 |
| Awake or somnolent | 24 (11 – 89) | 11 (5 – 26) | 0.152 |
| Stuporous or comatose | 10 (4 – 17) | 8 (6 – 12) | 0.562 |
| CPSE | 21 (11 – 55) | 11 (5 – 26) | 0.173 |
| GCSE | 10 (4 – 25) | 10 (2 – 14) | 0.825 |

Data in columns two and three are median weeks (interquartile range).

Abbreviations: cIVAD, continuous intravenous anesthetic drug; CPSE, complex partial status epilepticus; GCSE, generalized convulsive status epilepticus; STESS, Status Epilepticus Severity Score
